# Intrinsic dynamic behavior of enzyme:substrate complexes govern the catalytic action of β-galactosidases across clan GH-A

**DOI:** 10.1038/s41598-019-46589-8

**Published:** 2019-07-17

**Authors:** Rajender Kumar, Bernard Henrissat, Pedro M. Coutinho

**Affiliations:** 1 Architecture et Fonction des Macromolécules Biologiques, CNRS, Aix-Marseille Université, F-13288 Marseille, France; 2 USC1408 Architecture et Fonction des Macromolécules Biologiques, Institut National de la Recherche Agronomique, F-13288 Marseille, France; 3 Department of Biological Sciences, King Abdulaziz University, 23218 Jeddah, Saudi Arabia; 4 Polytech Marseille, Aix-Marseille Université, Marseille, France; 5 Present Address: Department of Clinical Microbiology, Umeå University, SE-901 85 Umeå, Sweden

**Keywords:** Computational biophysics, Computational biology and bioinformatics, Enzyme mechanisms

## Abstract

The conformational itineraries taken by carbohydrate residues in the catalytic subsite of retaining glycoside hydrolases (GHs), harness the link between substrate conformation and reactivity. GHs’ active sites may be described as a combination of subsites dedicated to the binding of individual sugar residues and to catalysis. The three-dimensional structure of GH:carbohydrate complexes has demonstrated that carbohydrate ring conformation changes in an ordered manner during catalysis. Here we demonstrate *in silico* that a link exists between subsite binding dynamics and substrate specificity for β-galactosidases from clan GH-A families GH1, GH2, GH35, GH42 and GH59. Different oligosaccharides were docked in the active site of reference β-galactosidase structures using Vina-Carb. Subsequent molecular dynamics (MD) simulations revealed that these enzymes favor a high degree of flexibility and ring distortion of the substrate the lytic subsite −1. Although the β-galactosidase families examined are structurally and mechanistically related, distinct patterns of ring distortion were unveiled for the different families. For β-galactosidases, three different family-dependent reaction itineraries (^1^*S*_3_ → ^4^*H*_3_^‡^ → ^4^*C*_1_, ^1,4^*B* → ^4^*H*_3_*/* ^4^*E*^‡^ → ^4^*C*_1_, and ^1^*S*_5_ → ^4^*E/* ^4^*H*_5_^‡^ → ^4^*C*_1_) were identified, all compatible with the antiperiplanar lone pair hypothesis (ALPH) for the hydrolysis of β-glycosides. This comparative study reveals the fuzzy character of the changes in carbohydrate ring geometry prior to carbohydrate hydrolysis.

## Introduction

β-Galactosidases (E.C. 3.2.1.23) catalyze the hydrolysis of terminal non-reducing end β-D-galactosyl residues found in oligosaccharides and glycoconjugates. Although the natural diversity of β-galactosidase substrates remains largely unexplored, reference substrates such as lactose and the convenient reporters *ortho*- and *para*-nitrophenol-β-D-galactosides (oNP-β-Gal and pNP-β-Gal) have been historically employed to assay β-galactosidase activity. β-Galactosidases can be obtained directly from microbial sources but also from plants and animals. This enzyme activity is heavily applied industrially to reduce the lactose content in dairy products, the transglycosylating properties of some enzymes being also employed for the production of galactooligosaccharides (GOS)^1–3^. Both free and immobilized enzyme preparations have been exploited in applications of biotechnological, medical, and analytical interest^4^.

β-Galactosidases are presently found in families GH1, GH2, GH35, GH42, GH50, and GH59 of the reference carbohydrate-active enzyme classification (described in the CAZy database, www.cazy.org)^5^, all included in clan GH-A. This clan groups together families of GHs that share the same global fold, catalytic mechanism and catalytic apparatus. Clan GH-A is the largest known GH clan and the corresponding enzymes are known for their action on equatorially-linked glycans^6^. Structurally, members of clan GH-A are characterized by a (β/α)_8_ barrel fold, a common retaining mechanism and a pair of conserved glutamic acid residues that act as an acid/base catalyst and a nucleophile. These two residues are present in structurally identical positions of the barrel. GH-A enzymes catalyze the hydrolysis not only of β-D-galactosides, but also of a variety of other equatorially-linked substrates such as β-D-glucosides, β-D-mannosides, β-D-xylosides or even α-L-arabinofuranosides and a-L-iduronides. Interestingly, the underlying active sites reveal a similar topology in the immediate vicinity of the anomeric carbon undergoing cleavage. The conserved pair of glutamate residues characteristic of clan GH-A, plays therefore identical roles for different substrates^7^.

Structural and kinetic data have been used to describe the binding of carbohydrates substrates into the enzyme active site and subsequent catalytic activity. The environment that accommodates a single carbohydrate residue following binding defines a subsite. Enzyme-carbohydrate complexes studied by X-xay crystallography, have gradually revealed details on the interactions of GHs with their substrates. By convention, the catalytic cleavage takes place between subsites −1 and +1 of a carbohydrate chain oriented from the non-reducing to the reducing end^8^. Dependent on active site geometry and the enzyme mode of action, the residues adjacent to the two reference catalytic subsites are incremented negatively or positively. The structure of several protein-carbohydrate complexes revealed a range of non-ground state ring geometries within subsite −1 of the active site^9,10^. These have been used to describe intermediate conformational forms along the reaction pathway and ultimately have led to the definition of key steps in the reaction pathway of retaining β-glycosidases^11–13^. Electrostatic, thermodynamic, and stereochemical considerations have all to be considered to understand the paths by which enzymes modify carbohydrate ring geometry. Ultimately, the conformational particularities of the cleaved residues affect the resulting reaction paths.

Exclusively present in clan GH-A, the widespread “classical” β-galactosidase activity has extensively been studied for decades. Abundant structural information on β-galactosidases is now available, revealing not only their apo form but also their complexes with substrates, reaction intermediates, products and substrate analogs. In order to better understand structure-functional relationships across different families within clan GH-A, we targeted β-galactosidases. In these exo-cleaving enzymes, the essential −1 subsite is generally complemented by a clearly defined +1 subsite, and may be complemented by adjacent subsites^14,15^.

Evidence clearly linking β-galactosidase specificity and reactivity is limited. Structural data on β-galactosidase complexes essentially detail interactions with the monomeric product or with the covalently linked reaction intermediate. Among the six families with this activity, only family GH2 yielded complexes with the substrates lactose and allolactose^16^, the latter being also a transglycosylation product. Notably, data on β-galactosidases complexed with carbohydrates bearing non-ground state ring geometries is very limited, remaining insufficient to identify the reaction itinerary (or deviations thereof) and other common aspects, if any, of the enzymatic action in the different enzyme families. State-of-the art molecular modeling approaches were here used to complement existing structural knowledge on β-galactosidases^5^ and to elucidate the dynamics of the enzyme:substrate interactions, whose subtleties are difficultly addressed by X-ray crystallography alone. A diverse dataset of β-galactosidases in clan GH-A was set using reference structural and biochemical data from the CAZy database in order to exploit their interaction with substrates *in silico*. We have used conventional flexible docking approaches aiming at discriminating virtually identical carbohydrates against different β-galactosidase structures. Designed to differentiate substrates by their binding affinity, and hence reflect specificity features, this approach had to be complemented by molecular dynamics (MD) so that carbohydrate ring geometry variants could be exploited in their full extent at subsite −1. The combined approach implemented here, was not only able to reveal details on induced fit in the formation of enzyme-substrate complexes but also the dynamical nature of the substrate distortion induced by the active site. The study unveils subsite −1 as considerably more dynamic than other subsites in these complexes. Surprisingly, the results yield different conformational itineraries for β-galactosidases belonging to different families within clan GH-A, all compatible with current views on the path to attain the transition state of retaining β-glycosidases in general.

## Computational Methodology

### β-Galactosidase structural and substrate diversity in clan GH-A

All clan GH-A reported PDB structures linked in the CAZy database^5^ were retrieved (December 2016) and clustered based on a 50% sequence identity criterion in order to limit structural redundancy. Subsequently, sequence independent structural alignments of the backbone atoms (N, C_α_, C, and O, extended to C_β_) were performed with MaxCluster^17,18^. Since all proteins in a clan are evolutionarily related, the structural pairwise alignments used for the comparisons are expected to reflect evolutionary distance. For the retained structures, a distance matrix was built based on 1-MaxSubAverage score for each structural pair. As the MaxSub scores are not symmetrical, two reciprocal MaxSub scores were calculated for each pair of protein structures and averaged to obtain a single value, MaxSubAverage. As 0 ≤ MaxSub ≤ 1 where 1 is “structural” identity, a structural distance for each pair was given as 1-MaxSubAverage and used to build a distance matrix. This matrix was used to infer a structure distance tree by the neighbor-joining method (http://www.trex.uqam.ca), subsequently visualized using iTOl (http://itol.embl.de).

The resulting tree was used to select β-galactosidase structures for subsequent studies. Experimentally reported information on β-galactosidases such as biological activity, active site and structural information was retrieved from the CAZy database^5^. β-Galactosidases have been reported in families GH1, GH2, GH35, GH42, GH50, and GH59, all from clan GH-A. However, family GH50 was not considered further because no representative structures for this activity exist at present. For the subsequent docking and MD simulation studies, we selected an ensemble of β-galactosidases that sampled the diversity found in the structural distance tree, and a diverse range of oligosaccharide substrates.

### Molecular docking

Molecular docking studies were performed using Vina-Carb, a variant of AutoDock Vina recently developed specifically for carbohydrate docking^19,20^. Vina-Carb takes in account specific features of carbohydrates during docking, including the glycosidic torsion angle preferences and the intramolecular energies of the glycosidic linkages. Thus, it combines the dual advantages of a large search space of Vina with a robust intramolecular energy evaluation of the glycosidic linkages when addressing carbohydrate-protein interactions.

### 3-D structures of substrates for docking

The substrates selected for docking initial were selected based on the reported substrates for the chosen enzymes (see below). They comprised the β-1-methyl derivatives of the monosaccharide galactose, of the disaccharides lactose, lactulose, β-1,3-lactosamine, β-1,3-galactodiose, β-1,6-galactodiose and of the trisaccharides cellotriose and β-1,4-galactotriose. To these, tetra- and pentasaccharides derivatives of xyloglucan were added to explore the binding of β-galactosyl residues found at branch termini by enzymes with known or putative activity on this polysaccharide. These regular, complex branched and carbohydrate derived ligand structures of known β-galactosidase substrates were generated using the online GLYCAM carbohydrate builder tool (http://glycam.org), where each structure was minimized and optimized using the GLYCAM_06j force field^21^, using the inbuilt SANDER simulation program^22^. A β-linked O-methyl group was systematically added to the reducing end of the saccharides, in order to better mimic longer substrates by limiting the impact of unfavorable H-bonding with free hemiacetal group. For long oligosaccharides, we retained the lowest energy conformer whenever more than one structure with different glycosidic torsions was suggested. For each substrate, the non-polar hydrogen atoms were merged to the adjacent heavy-atoms, and all possible rotatable bonds were defined using the AutoDock Tool (ADT)^23^.

### Enzyme structures and docking

Whenever possible, the enzyme structures selected for docking involved reference protein:carbohydrate complexes^5^. In order to prepare docking, we used the standard protocol of ADT. For each protein structure, the coordinates of all ions (except for conserved active site ions where needed), ligands, and water molecules were removed. Polar hydrogen atoms were added and non-polar hydrogens were merged to their adjacent heavy atoms for both the protein and the ligands. Finally, Kollman united atom charges and atom-type parameters were added for the protein while Gasteiger charges were added for each ligand. The docking reference grids were set to completely involve the active site, using the catalytic residues as a reference and overall active site topology for orientation. The top 30 docking conformations of each Vina-Carb simulation were retained for further analysis. Otherwise, all docking parameters were set to default values.

### Molecular Dynamics simulations

**Figure 1:**
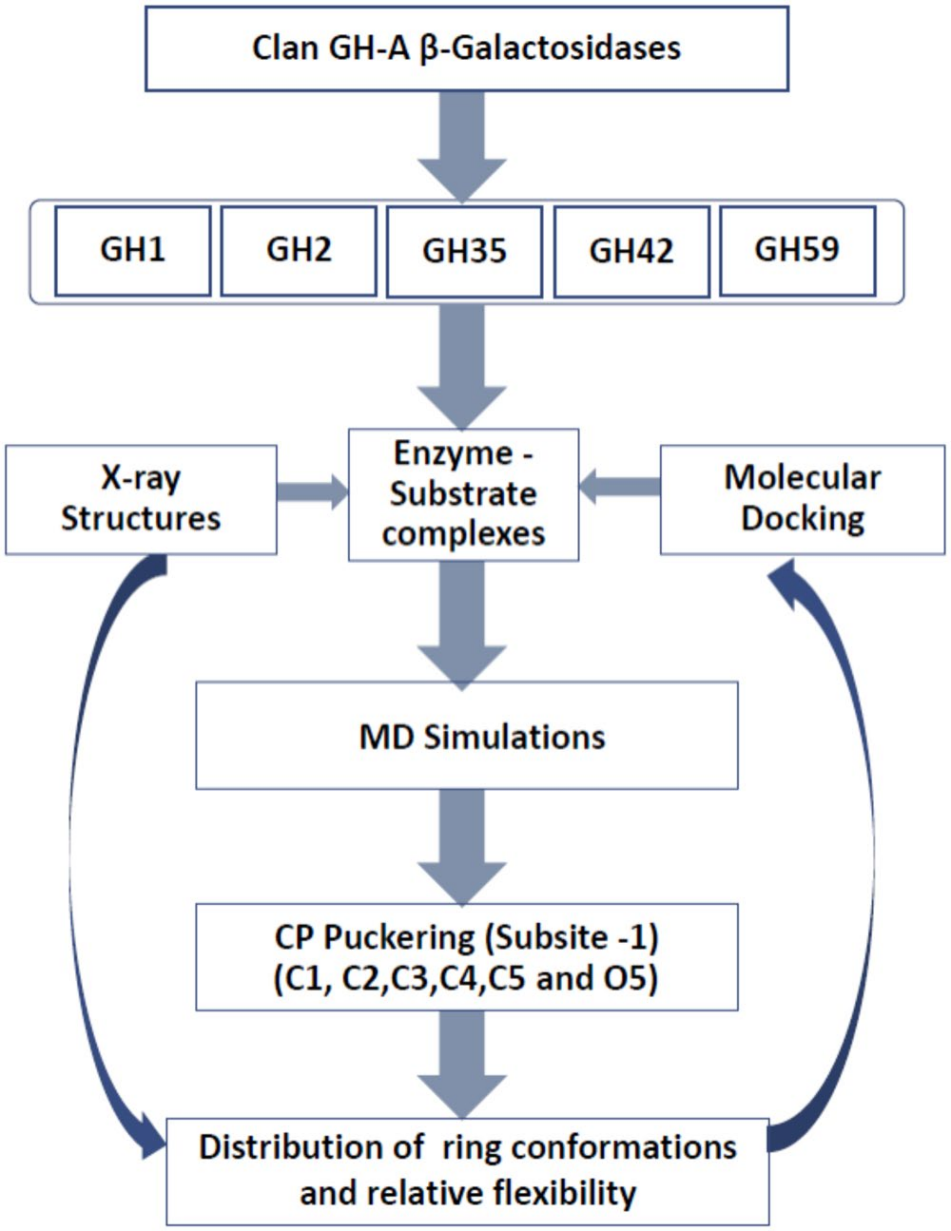
Flowchart of methodology adopted in this work.

Classical MD simulations of the enzyme-substrate complexes were performed using the AMBER 14.0 package^24^ where the GLYCAM_06j-1 and ff14SB force field parameters were applied for the carbohydrate ligands and for proteins, respectively^21,25^. For these simulations, the initial coordinates of each complex under study were selected from docking results using the following criteria: (i) the best dock score (ii) the superimposition with reference data, (iii) the best fit conformation of the substrate nonreducing end in subsite −1 and (iv) fundamental interactions with active site and nucleophilic and acid-base catalysis including appropriate distance from the sugar anomeric center. In the AMBER leap module, all necessary parameters were employed, considering the ionizable residues set at their default protonation states at a neutral pH value. The nucleophilic and acid/base residue side chains were assigned to a deprotonated and to a protonated state, respectively. The complexes were neutralized by adding Na^+^ or Cl^−^ ions and solvated using standard TIP3P model water molecules in a truncated octahedron box^26^ with a margin distance of 10 to 12 Å. The particle mesh Ewald method was used to account for long range coulombic interactions^27^, with a cut-off of 10.0 Å. The SHAKE algorithm^28^ restrained all atoms covalently bonded to hydrogen atoms, allowing for an integration time step of 2 fs. Periodic boundary conditions were applied to avoid edge effects. The solvent molecules and counter ions were minimized by 1,000 steps of steepest descent method followed by 500 steps of conjugate gradient method, while restraining the protein using a force constant of 2 kcal/mol Å^2^. The system was gradually heated from 0 to 300 K over a period of 50 ps and maintained at 300 K with a force constant of 2 kcal/mol Å^2^ on the complex. The system was then equilibrated to a free simulation for 0.5 ns. Finally, a production run of 20 ns was performed using the NPT statistical ensemble at 300 K with 1.0 atm pressure. This run was subsequently extended to 100 ns for several cases. Coordinate trajectories were recorded every 20 ps and analyzed using VMD 1.9.3^29^ and Chimera 1.9^30^. Ring conformation distributions of the carbohydrate residue at subsite −1 were estimated by the Cremer-Pople parameter calculator^31,32^. RMSD calculations were performed on the simulated complexes by steadily holding the protein heavy atoms during the alignment of the different time frames of the trajectory using Chimera version 1.9^30^ and the initial time frame as a reference. All water molecules and hydrogen atoms were ignored for these calculations. The RSMD data was plotted for the protein moiety, for the complete ligand scaffold, and for individual sugar residues. A flow chart of methodology used in this work done is given in Fig. 1.

## Results and Discussion

### Functional and structural diversity of β-galactosidases in clan GH-A

Clan GH-A is the largest of the GH clans, and represents an exceptional diverse evolutionarily-related group with a single fold and catalytic machinery^33^. At least 63 EC numbers (or distinct enzyme activities) are identified at present in the 22 families composing this clan, comprising both exo- and endo-cleaving enzymes (see supporting information SI.1). Although some families have only been characterized as exclusively exo- or endo-cleaving, the more extensively characterized families tend to have both types. The exceptional enzyme diversity in this clan can promote glycosidic bond cleavage at subsite −1 on at least 12 different glycosidic residues (see SI.1). These residues have topological resemblances that, when coupled with the identical catalytic machinery of the clan, lead to hydrolysis following Koshland’s double displacement mechanism^34^. Although some families like GH1 and GH5 have shown remarkable plasticity in their ability to act on a large variety of residues, one third of the families are relatively specific as they act only on a single type of substrates. β-Linked D-glucosyl, D-galactosyl, D-mannosyl and D-xylosyl residues constitute the major types of cleaved residues of clan GH-A enzymes. Families GH1 and GH5 are able to act on all those four residue types, while families GH2, GH17, GH26 and GH30 act only on three. Families GH35 and GH51, can cope with two different types, the remaining clan families having a single known activity at present. In total, ten clan GH-A families can promote the endo- or exo- degradation of saccharides containing β-galactosyl residues. The β-galactosyl-specific exo-acting enzymes will be here described generically as “β-galactosidase”.

**Figure 2:**
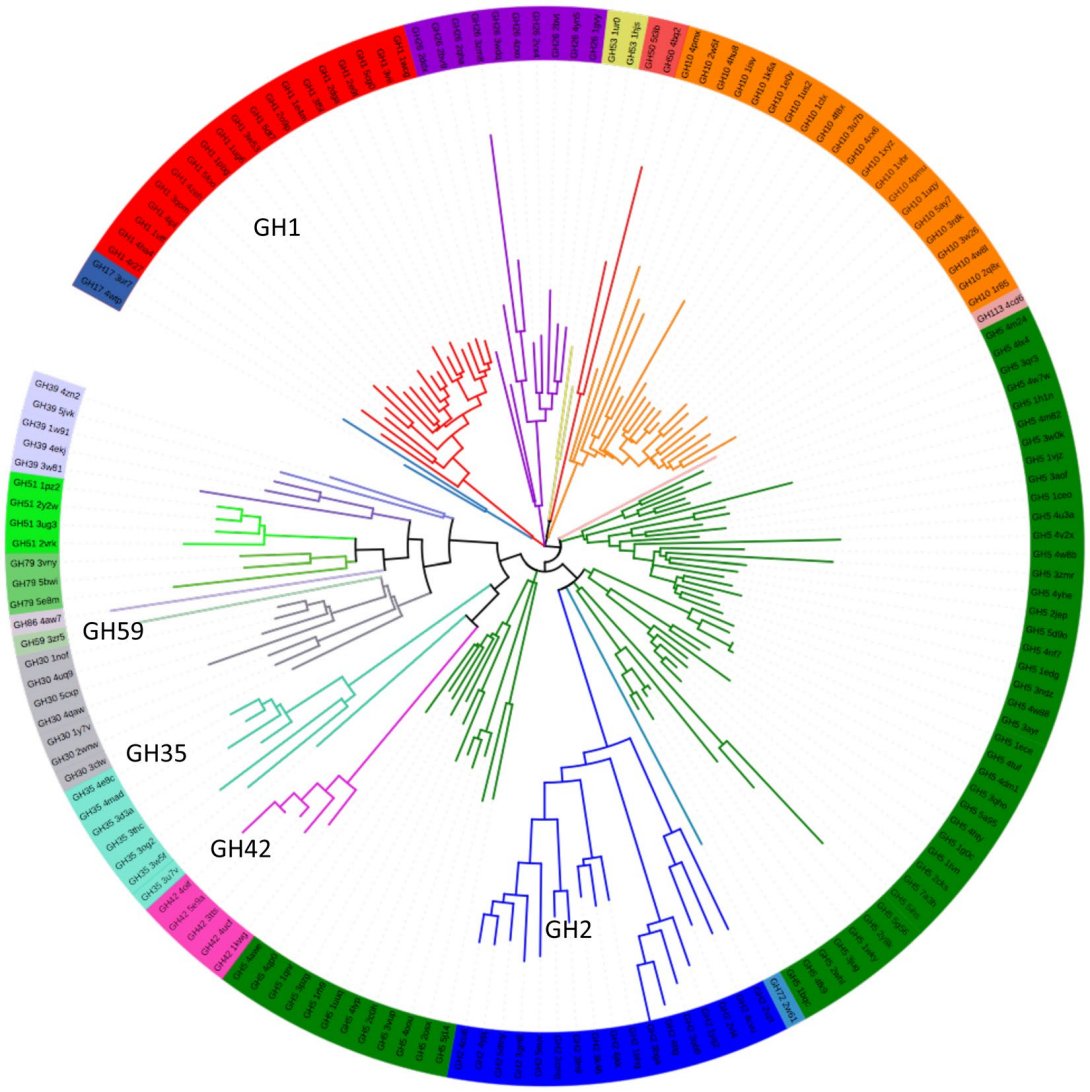
Clan GH-A structural diversity tree. Upon sequence clustering of all Clan-GH PDB entries on a sequence 50% identity basis, independent pairwise structural alignments of the backbone atoms (N, Cα, C, O and Cβ) were performed with MaxCluster for each structural pair. Structural distance 1-MaxSubAverage score was estimated from the average the non-symmetrical MaxSub scores obtained for each pair. A structural distance tree was determined by neighbor-joining, and visualized using iTOl (http://itol.embl.de). The leafs indicate the GH family and the PDB code. Leaf background and branch colors reflect the different Clan GH-A families with structural data. The clan families having β-galactosidases structures are indicated: GH1, GH2, GH35, GH42 and GH59.

A total of 255 PDB structures was initially retrieved from of all clan GH-A families. This number was reduced by applying a 50% sequence identity filter to obtain a final representative dataset of 160 distinct structures. These structures were compared pairwise to obtain the structural reference tree for clan GH-A described in Fig. 2. A significant number of families have a unique long branch at their base, a clear sign of their structural distinctiveness from the rest of the clan. This criterion clearly applies to 16 out of the 18 families with structural representatives in clan GH-A. A clear structural picture emerges in particular for families GH1, GH2, GH10, GH26, GH30, GH35, GH42, GH51 and GH79. Families GH17, GH50, GH53, GH59, GH72, GH86, and GH113 are presently represented only by one or two significantly distinct structures, leading to a clear positioning in the tree. Families GH5 and GH39 reveal however a structural sampling that is far from homogenous. For family GH5, the picture is particularly complex as its 52 representative structures were subdivided into three non-adjacent branches in the structural tree. Sequence analysis with large coverage had recently yielded on its subdivision into over 50 distinct subfamilies^35^. However, as only 14 subfamilies are structurally described in family GH5 and many of the structures have additional domains that affect the structural comparisons. Likewise in family GH39, two distinct structural groups are also due to additional domains attached to the common (β/α)_8_ barrel core.

The tree shows that most β-galactosidase families are structurally distinct, suggesting that this activity emerged repeatedly from a clan GH-A common β-glycosidase core. In this ensemble, families GH35 and GH42 appear as an exception, as they likely have split more recently from a β-galactosidase ancestor (see Fig. 2), as implied by recently described structural similarities^36^. Since the structural tree was calculated based on full PDB chains, rather than simply on the comparison of the (β/α)_8_ barrel, it indirectly reveals the influence of adjacent domains. Family GH2 enzymes in particular are known to integrate a number of associated domains that modulate its activity^33^. The presence and variability in domain numbers and size is responsible for the large branches for this family, illustrated in the distance tree. In spite of the structural diversity within family GH2, it is still represented in a unique branch. The long branches of family GH2 contrast with those depicted for family GH1. Family GH1 is particularly compact as its structures are only composed of the (β/α)_8_ barrel characteristic of the clan. Here however, β-galactosidases cannot be easily singularized as this enzyme activity is often associated with others in β-glycosidases.

### Michaelis-Menten [E.S] complexes: from carbohydrate docking to molecular dynamics

Carbohydrate substrate interactions with different β-galactosidase structures representative of clan GH-A were initially explored by a combination of molecular modeling and docking. The enzyme-substrate interactions targeted by our studies correspond to those expected in the Michaelis’ enzyme-substrate [E.S] complex. We assumed that this pre-transition state could be approached using molecular mechanics as the electronic and conformational distortions and the changes of critical bond lengths found in the subsequent ion-like transition states are expected to be close neighbors of ground-state electronic configurations^13^.

**Figure 3:**
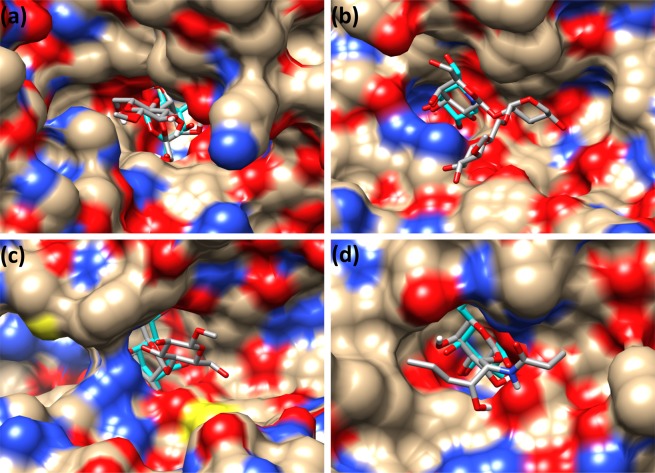
Best docked conformers of selected β-D-galactosides in representative β-galactosidase structures. Docking with Vina-Carb was contrasted with co-crystal ligands for β-galactosidases issued from different GH families: (**a**) 1-methyl-β-lactoside = DGalpb1–4DGlcp-OMe (GH1, PDB ID: 1UWR), (**b**) xyloglucan trisaccharide derivative (L-OMe) = DGalpb1–2DXylpba1–6DGlcp-OMe (GH35, PDB ID: 4DIJ), (**c**) 1-methyl-β-lactoside = DGalpb1–4DGlcp-OMe, (GH42, PDB ID: 3TTY) and (**d**) DGalp-Cerebroside-derivative with truncated aliphatic chains (GH59, PDB ID: 3ZR6). The co-crystal ligands are shown in cyan color.

We have docked reported disaccharide substrates described in reference enzyme:substrate complexes by the CAZy database^5^ or following the biochemical activity characterizations described in the literature for the selected enzymes. Molecular docking studies with Vina-Carb were able to provide initial data on the mode of binding between β-galactosidases and their true substrates. Best docked conformations and their molecular level interaction with the enzyme are illustrated for selected cases in Fig. 3(a–d).

The non-reducing-end of oligosaccharides with a terminal β-D-galactosyl-residue in the canonical ^4^*C*_1_ chair conformation could be docked optimally at subsite −1 in a limited number of cases (in families GH1, GH42, GH59 — RMSD <0.5 Å for the pyranose ring of galactosyl residues identified in reference complexes) while in others this was not possible (in families GH1, GH2 and GH35 — RMSD >0.5 Å for the pyranose ring). Thus, only in some cases the active site geometry allows the maximization of interactions, and therefore favors binding of the substrate deep into the active site. In family GH59, the active site pocket accommodates exclusively the β-D-galactosyl moiety at subsite −1, revealing an open active site where no obvious subsite can be identified that would directly interact with the adjacent sphingosine and fatty-acid tails. Reported data on ligand interactions is presently limited to a galactose-bound structure^37^. Docking was performed on this reference protein structure with substrate bearing a full length cerebroside moiety and on a truncated version. In both cases, the β-D-galactosyl moiety perfectly fitted the active site pocket (Fig. 3d), resulting in a near perfect superposition with crystallographic data (RMSD <0.5 Å for the pyranose ring).

Molecular modeling studies of carbohydrates and their derivatives in protein binding sites are often challenging due (i) to difficulties in estimating water-mediated interactions, (ii) to the multiple H-bonds of each of the many and inherently flexible terminal hydroxyl groups, (iii) to the contribution of stacking between protein aromatic residues and the hydrophobic surfaces of carbohydrate-rings, and (iv) to the energies associated with conformational changes involving glycosidic bonds. The molecular docking results alone are not totally satisfactory as the docked β-D-pyranose ring at subsite −1, did not always superpose with the reference data (in terms of RMSD, as mentioned before). The positive aspect is that all cases lead to docking poses where the β-D-galactosyl-non-reducing end residue can be found in the active site. In a large number of cases however, the fit to the catalytic pocket appears suboptimal since the β-D-galactosyl moiety does not enter deep enough in subsite −1. In these cases, the local H-bond networks −1 show significant differences to that identified in complexes with the product. Binding at subsites +1 or higher is severely affected by these dislocated poses.

The enzyme active site has evolved to accommodate non-ground states of the substrate corresponding to intermediate steps of the reaction. The formation of the enzyme-substrate complex is accompanied by conformational changes in the active site, both at the enzyme and substrate level. In our docking studies, the enzymes were treated as rigid entities and only the canonical ^4^*C*_1_ chair found in relaxed substrates was considered for the substrate. In what concerns substrate-ring distortion, only a few reference β-galactosidase complexes revealed conformations different from the ^4^*C*_1_ ground-state. For example, in family GH2 several enzyme:substrate complexes are found where the non-reducing end pyranose ring is in the ground-state conformation, including lactose (PDB:1JYN) and *N*-acetyl-lactosamine (PDB:4CUC), and with only one case where the complex with allolactose (PDB:4DUW) shows significant distortion of the sugar ring to a ^4^*H*_3_ conformation^38^. As carbohydrate rings are treated rigidly by current docking protocols, their distortion could be addressed by defining a representative universe of possible ring conformers prior to docking, as reported for cellobiose phosphorylase in family GH94^39^. Such constraints constitute however a serious limiting factor, as a large number of discrete ring conformers has to be generated, tested and analyzed. Ultimately the exact conformation of the ring cannot be determined *a priori*. Modeling was therefore extended from docking to MD simulations, where ring conformational space is not constrained.

### Ring flexibility and dynamics in clan GH-A β-galactosidases

The complexes obtained from our initial docking results were subjected to a dedicated MD approach in order to take in account the flexibility features of both protein and glycan partners during the formation of the enzyme-substrate complex. MD is expected to allow the exploration of ring distortion in conjunction with other conformational changes of both the protein and ligand, and to provide a better insight into the changes of the Cremer-Pople puckering conformation – describing the geometry of the pyranosyl ring – from initial binding to productive [E S] complex formation. For each protein-substrate complex, the distribution of the ring conformations were estimated from the trajectories obtained from productive runs lasting 20 or 100 ns. The ring geometry of the β-D-galactopyranosyl residue at subsite −1 was monitored after each of 1,000 frames of MD simulations. The distribution of calculated ring conformations is reported in Table 1.

**Table 1:** Relative puckering distributions at subsite −1 identified in clan GH-A β-galactosidases during MD simulations.

Family	PDB	Organism	Substrate	Time (ns)	^4^ *C*_1_	*E*_3_	^4^ *H*_3_	^4^ *E*	^4^ *H*_5_	*E*_5_	^O^ *H*_5_	*B*_3,O_	^1^ *S*_3_	^1,4^ *B*	^1^ *S*_5_	*B*_2,5_
GH1	1UWR	*Sulfolobus solfataricus P2*	DGalpb1–4DGlcp-OMe/Lac-OMe	20/100	**99.4/99.8**	−/−	0.1/<0.1	0.4/<0.1	0.1/<0.1	−/−	−/−	−/<0.1	−/<0.1	−/−	−/−	—
			DGlcpb1–4DGlcpb1–4DGlcpb-OMe	20	**98.2**	—	—	0.2	0.4	1.2	—	—	—	—	—	−/−
	5CG0	*Spodoptera frugiperda*	DGalpb1–4DGlcp-OMe/Lac-OMe	20/100	**90.8/88.0**	−/−	−/0.4	2.9/6.4	5.3/3.8	0.9/0.3	0.1/<0.1	−/−	−/0.3	−/0.7	−/−	—
GH2	1YJN	*Escherichia coli str. K-12*	DGalpb1–4DGlcp-OMe/Lac-OMe	20	**100**	—	—	—	—	—	—	—	—	—	—	—
	5LDR	*Paracoccus sp. 32d*	DGalpb1–4DGlcp-OMe/Lac-OMe	20	**98.0**	—	0.5	0.1	—	—	—	0.1	1.3	—	—	−/−
GH35	3OGR	*Trichoderma reesei*	DGalpb1–4DGlcp-OMe/Lac-OMe	20/100	**97.9/99.4**	−/−	−/<0.1	0.7/0.2	0.1/<0.1	−/−	−/−	−/−	1.0/0.2	0.3/<0.1	−/−	—
			DGalpb1–4DFruf-OMe	20	**99**	—	0.5	0.5	—	—	—	—	—	—	—	−/−
			DGalpb1–4DGalpb1–4DGalpb-OMe	20/100	**82.1/95.5**	0.2/<0.1	3/0.7	10.8/2.9	3.3/0.7	−/−	−/−	−/−	0.5/0.1	0.1/<0.1	−/−	−/−
	4MAD	*Bacillus circulans*	DGalpb1–3DGlcNAcp-OMe	20/100	6.4/22.6	−/−	7.8/8.2	13.9 /15.3	0.2/0.4	0.1/<0.1	−/−	−/<0.1	**37.2/31.2**	34.4/22.2	−/<0.1	−/−
	4E8C	*Streptococcus pneumoniae*	DGalpb1–3DGlcNAcp-OMe	20/100	**93.3/98.7**	−/−	3.4/0.7	2.1/0.4	−/<0.1	−/−	−/−	−/−	1.0/0.2	0.2/<0.1	−/−	−/−
GH42	3TTY	*Bacillus circulans*	DGalpb1–4DGlcp-OMe/Lac-OMe	20/100	**96.6/99.3**	−/−	0.9/0.2	2/0.4	0.5/0.1	−/−	−/−	−/−	−/−	−/−	−/−	—
	4OIF	*Geobacillus stearothermophilus*	DGalpb1–4DGlcp-OMe/Lac-OMe	20	**99.6**	—	—	0.1	0.3	—	—	—	—	—	—	—
	4UNI	*Bifidobacterium animalis*	DGalpb1–3DGalp-OMe	20	**100**	—	—	—	—	—	—	—	—	—	—	—
			DGalpb1–6DGalp-OMe	20	**100**	—	—	—	—	—	—	—	—	—	—	—
	4UCF	*Bifidobacterium bifidum S17*	DGalpb1–4DGlcp-OMe/Lac-OMe	20	**99.8**	—	—	0.2	—	—	—	—	—	—	—	1.0/0.2
	1KWG	*Thermus sp. A4*	DGalpb1–4DGlcp-OMe/Lac-OMe	20/100	**75.2/89.1**	—	0.7/0.8	8.4/4.7	5.5/2.6	0.2/0.2	—	−/<0.1	−/0.2	2.5/0.7	6.5/1.52	—
GH59	3ZR6	*Mus musculus*	DGalpb1-OMe	20	**95.3**	—	0.1	1.9	2.7	—	—	—	—	—	—	

Percentage of each ring conformer shape attained by the non-reducing end residue at subsite −1 sampled in 1000 or 5000 time steps throughout 20 or 100 ns MD simulations, respectively, for different carbohydrate substrates against targeted β-galactosidases from clan GH-A. The different enzymes are identified by a reference PDB code and by species. The non-reducing end residue is a β-D-galactopyranosyl residue, excepting for a single substrate were it is a β-D-glucopyranosyl residue, shown in bold. The most abundant ring conformer for each MS simulation is highlighted in bold.

The substrates of the studied β-galactosidases adopted different dominant ring conformations during the formation of the Michaelis complex. Large variations were observed at subsite −1 in a number of cases, contrasting with the absence of changes observed for simulations of 1-methyl-β-D-galactopyranoside in solution using the same MD protocol (data not shown). Free energy calculations for a retaining β-glycosidase have previously shown that the active site induces alternative ring conformations that are significantly more stable than the ground-state conformer found in solution^40^. The changes observed dependent on the GH family, on the protein targeted, on its relative flexibility and dependence on active site ions, and finally on the docked substrate. Three ring conformations, namely ^4^*H*_3_, ^4^*E* and ^4^*H*_5,_ were observed for the terminal β-D-galactopyranosyl residue in the simulations of practically all β-galactosidases. Ring conformers related to ^4^*H*_3_ and to ^4^*E* have been previously identified using transition analogs in family GH2 *E. coli* LacZ (PDB:1JZ6 and 3VD7), and were later corroborated by QM approaches^16,41^.

Interestingly, most simulated complexes of family GH1, GH35 and GH42 β-galactosidases with their substrates yielded prominent deviations from the canonical ^4^*C*_1_ chair for the β-D-galactosyl residue at subsite −1. In these families, other ring conformational states at subsite −1 add up to about 10% or more of the trajectory frames (see Table 1). In family GH2, only the structure of the β-galactosidase from the psychrophile *Paracoccus sp. 32d* yielded simulations with distorted conformations, contrasting with all the enzyme:substrate complexes from mesophiles. The relatively higher degree of flexibility of the psychrophile enzyme could partially explain the different behavior, but other factors like the presence of ions and the mobility of active loops may negatively affect enzyme-substrate interactions. The lactose to allolactose cycle observed for the classical *E. coli* β-galactosidase^42^ may require specific dynamic features when compared to simple hydrolysis, with possible impact on the simulations. Slow acting hydrolases would then exhibit lower levels of distortion, lowering the probability that catalysis takes place.

**Figure 4:**
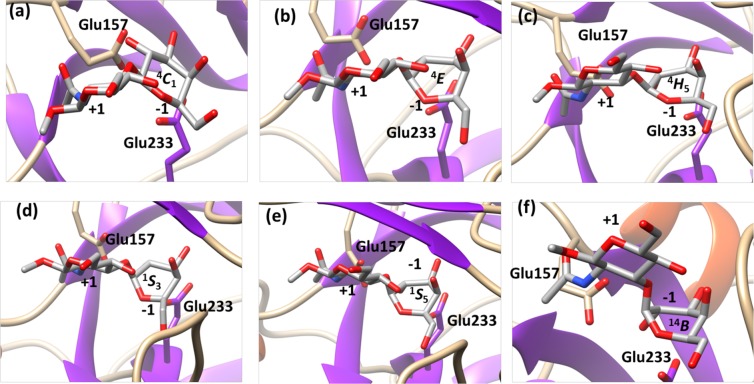
Puckering changes observed at subsite −1 during MD simulations in family GH35. Selected conformational states at subsite −1 of β-1,3-LacNAc-OMe = DGalpb1–3DGlcNAcp-OMe during MD analysis (GH35, PDB ID: 4MAD).

**Figure 5:**
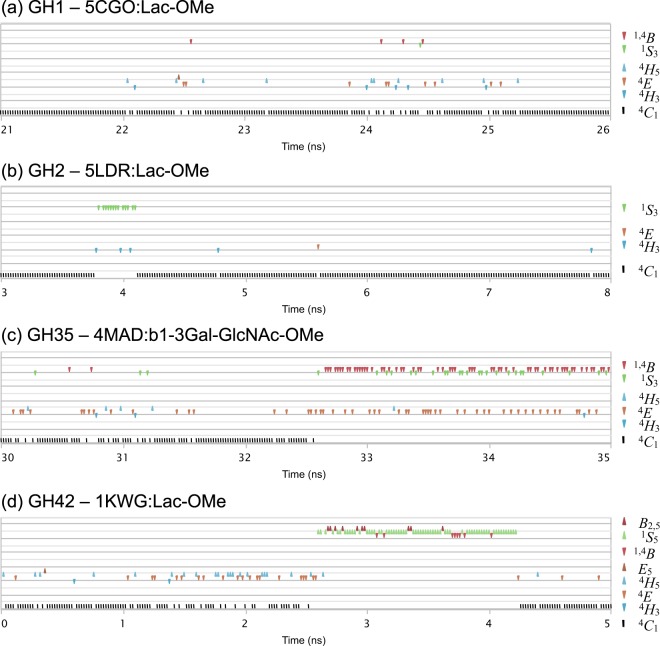
Puckering variation with time observed at subsite −1 during MD simulations in families GH1, GH2, GH35 and GH42. Selected 5 ns time segments that display conformational itineraries going from basal ^4^*C*_1_ up to equatorial boat (*B*) and skew-boat (*S*) states are observed: (**a**) 1-methyl-β-lactoside = Lac-OMe (GH1, PDB ID: 5CGO); (**b**) 1-methyl-β-lactoside = Lac-OMe (GH2, PDB ID: 5LDR); (**c**) β-1,3-LacNAc-OMe (GH35, 4MAD); (**d**) 1-methyl-β-lactoside = Lac-OMe (GH42, PDB ID: 1KWG).

Using the Mercator projection map of the Cremer-Pople puckering sphere for ring conformation description, the extent of the changes observed in the simulations correlates with the “equatorial” levels of distortion – boat (*B*) or skew-boat (*S*) ring configurations – of the residue found in subsite −1, in the vicinity of the skew-boat ^1^*S*_3_ local pucker energy minimum of β-D-galactopyranose^43^. A number of complexes in families GH1, GH2, GH35, and GH42 attained “equatorial” distortions during the simulations, yielding at least one of the ^1^*S*_3_, ^1,4^*B* or ^1^*S*_5_ ring geometries. One of the GH2 20 ns simulations yielded a ^1^*S*_3_ skew-boat conformer, while both ^1^*S*_3_ skew-boat and ^1,4^*B* boat conformers where obtained in a single long 100 ns run in family GH1 but in all four independent short 20 ns runs simulations in family GH35. One of our simulations in family GH42 explored all *B*_3,O_, ^1^*S*_3_, ^1,4^*B*, ^1^*S*_5_ and *B*_2,5_ conformers during a 100 ns simulation, although the first two only marginally and only after the initial 20 ns. More details about the ring geometries reached during the simulation studies and how their vary in time are shown in Figs 4, 5 and Supporting Information SI.2 and SI.3.

These variations of ring forms observed for β-galactosidases from different families do not lead to a simple and common explanation for all observations. Generally, the basal chair ^4^*C*_1_ conformer can interchange with a number of closely-related intermediate envelope (*E*) and half-chair (*H*) conformers throughout the simulations. The extent of this interchange during the simulations varies from only a few observations to a more frequent occurrence. For the vast majority of cases attaining an “equatorial” conformation, reaching intermediate envelope or half-boat forms is a pre-requisite. Afterwards, the equatorial conformation may be attained in a few bursts or during more extended periods. Although 100 ns simulations tend to yield a more diverse range of ring conformational states at subsite −1 than 20 ns runs, longer simulation periods tend to be less productive. With the exception of a single family GH1 that revealed its full conformational changing potential at later times, the 20 ns simulations were enough to attain meaningful conformational variability. Given the major impact of protein intrinsic properties on the simulations, we presently esteem that the shorter simulations are generally sufficient to reveal significant dynamic properties, longer studies bringing more statistically supported results.

Significantly, the observed conformational changes tend to correlate with the reaction itineraries proposed for retaining β-glycopyranosidase action according with the antiperiplanar lone pair hypothesis (ALPH) described by Nerinckx *et al*.^13^. For retaining β-glycosidases, the ALPH itineraries are described in a sequence of conformational steps where a skew-boat-like pre-transition state precedes a half-chair or envelope transition state and finally a ground-state ^4^*C*_1_ glycosyl-enzyme intermediate. The conformer distributions obtained for families GH1, GH2, GH35 and GH42, are clearly compatible with the proposed hypothesis, where the reaction itineraries would be ^1^*S*_3_ → ^4^*H*_3_^‡^ → ^4^*C*_1_ for GH2, ^1,4^*B* → ^4^*H*_3_*/*^4^*E*^‡^ → ^4^*C*_1_ for GH1 and GH35, and ^1^*S*_5_ → ^4^*E/*^4^*H*_5_^‡^ → ^4^*C*_1_ for GH42. As mentioned above, such mechanism is compatible with the transition state conformer ^4^*H*_3_/^4^*E*^‡^ described for β-galactosidase in family GH2^38^. No ALPH-compatible ring geometry distributions could be obtained for family GH59, as our simulation was unable to reach equatorial conformational states. For this family, no subsite +1 could be defined. Here, the absence of a lipidic support constraining the substrate at the subsite +1 could influence the positioning and by consequence the conformational behavior at subsite −1.

No significant ring geometry changes were obtained consistently in a number of our experiments. For instance, no changes beyond the regular ^4^*C*_1_ chair could be observed for the ring at subsite −1 for the GH2 *E. coli* LacZ, for GH2 *Streptococcus pneumoniae* BgaA (SpBgaA) and for GH42 *Bifidobacterium animalis* Gal42A (BlGal42A) β-galactosidases. Different factors may explain these negative results. In family GH2, the difficulties for the substrate to enter deeper into the active site observed during MD could be due to the presence of metal ions in the immediate vicinity of subsite −1 or to the open position of an active site loop influencing subsite +1. For family GH42, our docking results corroborated what was previously described^43^ for the interaction with β-1,3- and β-1,6-linked β-galactobiosides, with a clear fit of these disaccharides in the enzyme active site. However, no ring conformational changes at subsite −1 were observed. As for GH2, significant conformational changes in active site loops of BlGal42A β-galactosidase affect binding, but the necessary large scale movement of these loops induced by ligand binding could not be observed during the short time MD simulations used in this study. Intrinsic protein, and possibly substrate, features rather than computation time may thus represent limiting factors for successful ring conformational exploration through MD simulation.

**Figure 6:**
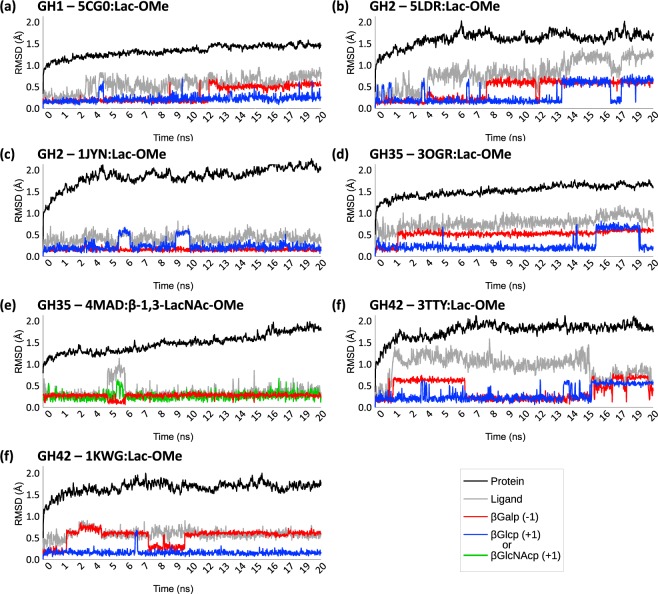
Relative flexibility of carbohydrate residues bound at subsites −1 and +1 during 20 ns MD simulations. The relative flexibility the residues found at subsites −1 (red) and +1 (dark blue) are contrasted with those of the full ligand (silver) and of the full protein (black) throughout 1,000 time steps of independent 20 ns MD simulations: (**a**) 1-methyl-β-lactoside = Lac-OMe (GH1, PDB ID: 5CGO); (**b**) 1-methyl-β-lactoside = Lac-OMe (GH2, PDB ID: 5LDR); (**c**) 1-methyl-β-lactoside = Lac-OMe (GH2, PDB ID: 1JYN); (**d**) 1-methyl-β-lactoside = Lac-OMe (GH35, PDB ID: 3OGR); (**e**) β-1,3-LacNAc-OMe (GH35, 4MAD); (**f**) 1-methyl-β-lactoside = Lac-OMe (GH42, PDB ID: 3TTY); (**g**) 1-methyl-β-lactoside = Lac-OMe (GH42, PDB ID: 1KWG).

When glycans bind to proteins, large internal rotations of the glycan moiety are generally reduced. In order to distinguish the dynamic nature of substrate behavior, the RMSD fluctuations of individual carbohydrate residues occupying the catalytic subsites were measured using the full protein and the initial time frame as references (see Method). In general, higher fluctuations of the carbohydrate heavy atoms (given in RMSD terms) are observed at subsite −1 than at subsite +1 (see Fig. 6a–e). For the carbohydrate residue at subsite −1, major changes are observed on the relative positions of the anomeric carbon and neighboring atoms. Such restrictions orient conformational variation towards specific “equatorial” and intermediate ring conformers of the CP puckering sphere. The only exceptions to this behavior occur when the ring conformation is either unable to evolve from the initial ^4^*C*_1_ chair (see Fig. 6c) or when on significant conformational changes occur prior to the start of the productive run impacting the reference state (see Figs 6e and SI.3). This suggests that the levels of sugar ring distortion and of fluctuation in RMSD terms of the residue found at subsite −1 are connected. This remarkably spacious subsite^13^ sets an environment that locally promotes the flexibility of the substrate, orienting the changes toward viable distorted ring conformations so that catalysis can occur.

### Selectivity and subsite inference from β-galactosidase structures

We have studied a representative set of clan GH-A β-galactosidases using substrates reported in the literature as our primary targets. In our studies, we used 1-methyl-β-lactoside whenever the activity on lactose was experimentally described as our main reference for studying enzyme:substrate complexes *in silico*. Binding in different subsites plays an important role in substrate specificity. If the presentation of the substrate at subsite −1 is essential for catalysis, we have studied binding *in silico* not only in subsites +1, but equally for subsites +2 and beyond whenever relevant.

In family GH35, three different substrates were tested by docking and MD against β-galactosidase Bga1 (also described as galacto-β-galactanase) from *Trichoderma reesei* (TrBga1), namely methyl-β-1,4-galactotrioside, 1-methyl-β-lactoside and 1-methy-β-lactuloside. TrBga1 active site is versatile in its ability to productively accommodate the β-linked glycosidic bonds with galactosyl, glucosyl, and fructosyl moieties present each of these substrates, respectively, presenting diverse relative orientation of the sugar residue at subsite +1. Interestingly, all substrates yielded relevant changes in the ring geometry of the non-reducing end β-D-galactosyl residue at subsite −1. In addition to the regular ^4^*C*_1_ chair, up to six and four other conformational states were attained during MD simulations for the former two substrates, but only two for the latter. Notably, the more extended changes in ring geometry observed for β-1,4-galactotrioside and the associated identification of a specific subsite +2 emphasize the pertinence of TrBga1 in the degradation of β-galactans^44^.

In family GH1, the β-glycosidase SsLacS was also studied in its interaction with two substrates: 1-methyl-β-lactoside and methyl-β-cellotrioside, the latter representing the reported activity against β-1,4-linked cellooligosaccharides^45^. Both substrates are well accommodated in the active site upon docking, MD revealing that distortions of the sugar residue at subsite −1 can take place for both terminal β-D-galactosyl and β-D-glucosyl residues. SsLacS active site was however apparently more extended and able to accommodate larger oligosaccharides.

**Figure 7:**
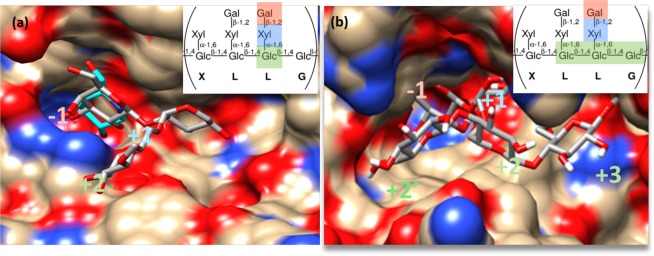
Docking of xyloglucan fragments against two β-galactosidases from different GH families. Best docked conformations following explorative search with xyloglucan fragments of different size against two different β-galactosidase structures: (**a**) xyloglucan side chain trisaccharide (GH35, PDB ID: 4DIJ); (**b**) branched xyloglucan side chain pentasaccharide (GH1, PDB ID: 1UWR). The different docked xyloglucan fragments are highlighted in color in the upper right corner. The colors reflect the putative enzyme carbohydrate residues whose corresponding subsites are depicted. The letters below the different residues of xyloglucan reflect the reference branch nomenclature for xyloglucan and derived oligosaccharides.

As mentioned earlier, β-galactosidases usually rely on the standard subsites −1 and +1, while some can extend the interactions to subsites +2 and beyond. Extended active sites with additional subsites may increase binding affinities and reveal a finer selectivity. Among all the explored structures, β-galactosidase Bgl35A from *Cellvibrio japonicus* (CjBgl35A) showed hydrolytic activity on xyloglucan and on different xyloglucan-derived oligosaccharides with a terminal β-galactosyl residue capping in its side chains^15^. Therefore, we performed docking experiments with short variants of these oligosaccharides against CjBgl35A. These selected oligosaccharides have a terminal galactosyl residue and contain from 1 to 3 glucosyl residues corresponding to the β-glucan-backbone, and can be described as L-Me, LG-Me, and GLG-Me using the standard xyloglucan nomenclature (see Fig. 7) and where -Me represents a β-O-linked methyl residue at the reducing end. Docking clearly confirms the ability of CjBgl35A to envelop the side chain with the α-xylosyl unit perfectly positioned in the immediate vicinity of subsite −1, confirming the location suggested for subsite +1^15^. The positioning of the β-glucan-backbone of xyloglucan can also be inferred, particularly at subsite +2. Overall, the galactosyl and xylosyl moieties of xyloglucan appear near the surface of CjBgl35A, suggesting a large degree of flexibility in the interaction with different variants of xyloglucan and derived saccharides. For more details on the initial subsites see Fig. 7a.

The deep active site the GH1 β-glycosidase SsLacS^14^ is compatible with extended interactions with long substrates. In family GH1, deep active site pockets have already been experimentally described for the interaction of rice β-glucosidase (PDB: 3F5K, 3SCT, 4QLK, etc.) with cellotetraose^46^. By analogy, we explored by docking the interaction of xyloglucan-derived oligosaccharides with SsLacS, as done above with CjBgl35A. These experiments defined subsites −1 to +2 for xyloglucan side chains (exemplified in Fig. 7). The β-1,4-glucosyl residues of the backbone bind in a linear manner transversely to the side chain (more details are given in Fig. 7b). The comparison of the SsLacS active site with that of CjBgl35A, results in a common accommodation of the xylosyl moiety. However, the GH1 structure reveals a deeper active site where positive subsites can be more easily defined. Its active site topology contrasts with the more exposed site of CjBgl35A, suggesting that SsLacS interactions with xyloglucan could be stronger and more selective. Relevantly, the α-xylosidase XylS from *Sulfolobus solfataricus* (SsXylS) is able to act on xyloglucan derivatives^14^, suggesting that the SsLacS and SsXylS enzymes could have a complementary action in xyloglucan degradation.

**Figure 8:**
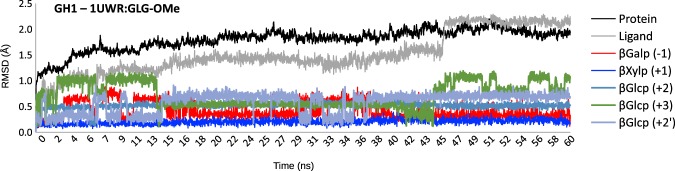
Relative flexibility at different subsites of bound residues from a xyloglucan-derived pentasaccharide against *S. solfataricus* β-glycosidase LacS during a MD simulation. The relative flexibility of the different carbohydrate residues depicted in Fig. 7b are shown for the residues at positions −1 (red), +1 (dark blue), +2 (steelblue), +3 (green) and +2’ (light slategray) and contrasted with those of the full ligand (silver) and of the full protein (black) throughout 3,000 time steps of a 60 ns MD simulation.

In order to validate the subsites identified upon docking, we carried out an extended MD simulation starting from the SsLacS:GLG-Me complex. Only minor ring conformation changes occurred for the β-D-galactosyl residue at subsite −1 during a 60 ns simulation. The α-xylosyl residue bound at subsite +1 remained in place throughout the simulation (Fig. 8). Interestingly, MD revealed that ring distortions occurred in the α-D-xylopyranosyl and β-D-glucopyranosyl residues bound at subsites +1 and +2, respectively. These results imply that ring flexibility may have an impact far from the cleavage point.

## Conclusions

The commonalities in structure and in biochemical behavior of enzymes belonging to the same clan provide predictive power on the mechanistic properties of putative proteins found in large datasets. The conserved protein structure and catalytic machinery within clan GH-A have evident plastic properties that allowed to generate an extraordinary diversity of enzymes able to cleave equatorially-linked glycans either in an exo- or in an endo-manner. In both cases, substrate distortions have been observed at subsite −1. The sugar ring at this subsite is then perfectly poised for the subsequent catalytic step. Distortion of the carbohydrate ring at subsite −1 thus contributes to the decrease of the activation energy and thus facilitates the cleavage of the glycosidic bond.

In this study, we investigated *in silico* the plasticity of interaction of clan GH-A β-galactosidases with known substrates. For a specific set of reference substrates, docking of substrates in enzymes and subsequent MD simulations of productive modes explored the path towards the Michaelis complex. The combination of these two approaches allowed (i) the definition of the subsites −1 and +1, and even infer higher order subsites when relevant, and (ii) the observation of dynamical features required for catalysis. Dynamics of the enzyme:substrate complex revealed to be a crucial factor for tuning the ring conformation of the β-galactopyranosyl residue at subsite −1 in order to attain the pre-transition state. Complementing the static views yielded by state-of-the-art protein crystallography, the enzyme active site appears here as a dynamic environment that promotes and orients substrate distortion in order to facilitate catalysis. Interestingly, our results do not point to a single ring conformer but to a cloud of different closely-related interchanging conformers. Rather than favoring single intermediate forms, the results suggests reaction paths with fuzzy conformational itineraries. Overall, the MD trajectories correlate with different predicted conformers proposed by the ALPH theory for the degradation of β-glycosides. In this way, the cleavage of β-galactosyl residues appears compatible with different reaction paths, namely the paths ^1^*S*_3_ → ^4^*H*_3_^‡^ → ^4^*C*_1_ for family GH2, ^1,4^*B* → ^4^*H*_3_*/*^4^*E*^‡^ → ^4^*C*_1_ for families GH1 and GH35, and ^1^*S*_5_ → ^4^*E/*^4^*H*_5_^‡^ → ^4^*C*_1_ for family GH42. For the studied case in family GH59, no skew-boat like distortions could be identified in useful MD simulation time. Independently of the family, transition-state-like ^4^*H*_3_ and ^4^*H*_5_ conformations were reached much more frequently than the above mentioned “equatorial” states during the simulations, leaving open the question for the requirement of ALPH-compliant itineraries in general. Are these itineraries required to simply obtain an appropriate geometry of interaction for catalysis? Or is there an associated inertia of the enzyme:substrate complex facilitating the configurational transition?

For practical purposes, the exploration of the universe of ring puckering conformers pertinent for enzyme-substrate interactions may facilitate the choice of a limited set of conformers for docking studies against a given enzyme structure. An analogous collection of conformers has been applied to study the interaction of β-glucose with cellobiose phosphorylase^39^. Rather than considering all the canonical ring conformers, only those pertinent for the given reaction mechanisms should be used. In the absence of experimental data, future docking studies involving β-galactosidases should then consider ring geometries representing all three itineraries identified in this study. Such distorted ring geometries may be required for the obtention of productive docking for some exo-acting glycosidases. Similar considerations will be certainly necessary for docking studies against endo-acting glycosidases, since long active sites are often incompatible with the positioning of non-distorted forms across the catalytic site. In all cases, MD should complement the docking studies to reveal the different ring forms tolerated by the enzyme active site and ideally relate these to the reaction configuration and itineraries.

Our studies suggest that the intrinsic dynamics of the enzyme:substrate complex promotes conformational instability of the carbohydrate residue present at subsite −1, orienting its ring toward geometries that increase the probability of the reaction to occur. For any given glycosidase, carbohydrate resemblances in terms of shape, glycosidic-bond connectivity and ring conformational properties of residues involved broaden the range of possible substrates, as long as a productive fit and required dynamics at subsite −1 remain possible. By allowing an extra degree of tolerance in enzyme-substrate interactions, the observed fuzziness of the ring conformational itineraries at the catalytic site is also likely at the origin of enzyme promiscuity, leading to the emergence of multiple enzyme activities in glycosidase families.

## Supplementary information

Additional information

## Data Availability

The datasets generated during and/or analysed during the current study are available from the corresponding author on reasonable request.
