# Supplementary material for: Intrinsic dynamic behavior of enzyme:substrate complexes govern the catalytic action of β-galactosidases across clan GH-A
Source: Sci Rep. 2019 Jul 17;9:10346. doi: 10.1038/s41598-019-46589-8 (PMC6637243; doi:10.1038/s41598-019-46589-8)

Additional information

Intrinsic dynamic behavior of enzyme:substrate complexes govern the catalytic action of β-galactosidases across clan GH-A

Rajender Kumar[1,2,5], Bernard Henrissat[1,2,3] & Pedro M. Coutinho[1,2,4,*]

[1]Architecture et Fonction des Macromolécules Biologiques, CNRS, Aix-Marseille Université, F-13288 Marseille, France.
[2]USC1408 Architecture et Fonction des Macromolécules Biologiques, Institut National de la Recherche Agronomique, F-13288 Marseille, France.
[3]Department of Biological Sciences, King Abdulaziz University, 23218 Jeddah, Saudi Arabia.
[4]Polytech Marseille, Aix Marseille Université, Marseille, France.
[5]Present Address : Department of Clinical Microbiology, Umeå University, SE-901 85 Umeå, Sweeden
*Correspondence: pedro.coutinho@univ-amu.fr

SI.1 Reported different GH activities of Clan GH-A in the CAZy database (>69 distinct EC numbers; >15 different residues at cleavage site). The major enzyme actives and type of cleavage found in each family are highlighted in bold. The enzyme activities studied in each family, namely β-galactosidase or β-galactocerebrosidase, are underlined in every family.

Family	Activities	Cleavage Residue (-1)	Type of Cleavage
**GH1**	**β-glucosidase** (EC 3.2.1.21); **<u>β-galactosidase</u>** (EC 3.2.1.23); **β-mannosidase** (EC 3.2.1.25); β-glucuronidase (EC 3.2.1.31); β-xylosidase (EC 3.2.1.37); β-D-fucosidase (EC 3.2.1.38); phlorizin hydrolase (EC 3.2.1.62); exo-β-1,4-glucanase (EC 3.2.1.74); 6-phospho-β-galactosidase (EC 3.2.1.85); 6-phospho-β-glucosidase (EC 3.2.1.86); strictosidine β-glucosidase (EC 3.2.1.105); lactase (EC 3.2.1.108); amygdalin β-glucosidase (EC 3.2.1.117); prunasin β-glucosidase (EC 3.2.1.118); vicianin hydrolase (EC 3.2.1.119); raucaffricine β-glucosidase (EC 3.2.1.125); thioglucosidase (EC 3.2.1.147); β-primeverosidase (EC 3.2.1.149); isoflavonoid 7-O-β-apiosyl-β-glucosidase (EC 3.2.1.161); ABA-specific β-glucosidase (EC 3.2.1.175); DIMBOA β-glucosidase (EC 3.2.1.182); β-glycosidase (EC 3.2.1.-); hydroxyisourate hydrolase (EC 3.-.-.-)	β-D-Glcp; β-D-Galp; β-D-Manp; β-D-GlcAp; β-D-Xylp; β-D-Fucp; β-D-6PGalp; β-D-6PGlcp	**Exo**/Endo
**GH2**	**<u>β-galactosidase</u>** (EC 3.2.1.23) ; β-mannosidase (EC 3.2.1.25); β-glucuronidase (EC 3.2.1.31); α-L-arabinofuranosidase (EC 3.2.1.55); mannosylglycoprotein endo-β-mannosidase (EC 3.2.1.152); exo-β-glucosaminidase (EC 3.2.1.165)	β-D-Glcp; β-D-Galp; β-D-Manp; α-L-Araf; β-D-GlcNp	**Exo**/Endo
**GH5**	**endo-β-1,4-glucanase / cellulase** (EC 3.2.1.4); endo-β-1,4-xylanase (EC 3.2.1.8); β-glucosidase (EC 3.2.1.21); **β-mannosidase** (EC 3.2.1.25); β-glucosylceramidase (EC 3.2.1.45); glucan β-1,3-glucosidase (EC 3.2.1.58); **licheninase** (EC 3.2.1.73); exo-β-1,4-glucanase / cellodextrinase (EC 3.2.1.74); glucan endo-1,6-β-glucosidase (EC 3.2.1.75); mannan endo-β-1,4-mannosidase (EC 3.2.1.78); cellulose β-1,4-cellobiosidase (EC 3.2.1.91); steryl β-glucosidase (EC 3.2.1.104); endoglycoceramidase (EC 3.2.1.123); **chitosanase** (EC 3.2.1.132); β-primeverosidase (EC 3.2.1.149); **xyloglucan-specific endo-β-1,4-glucanase** (EC 3.2.1.151); endo-β-1,6-galactanase (EC 3.2.1.164); hesperidin 6-O-α-L-rhamnosyl-β-glucosidase (EC 3.2.1.168); β-1,3-mannanase (EC 3.2.1.-); arabinoxylan-specific endo-β-1,4-xylanase (EC 3.2.1.-); mannan transglycosylase (EC 2.4.1.-)	β-D-Glcp; β-D-Galp; β-D-Manp; β-D-Xylp; β-D-GlcNp;	Exo/**Endo**
**GH10**	**endo-1,4-β-xylanase** (EC 3.2.1.8); endo-1,3-β-xylanase (EC 3.2.1.32); tomatinase (EC 3.2.1.-); xylan endotransglycosylase (EC 2.4.2.-)	β-D-Xylp	**Endo**
**GH17**	**glucan endo-1,3-β-glucosidase** (EC 3.2.1.39); glucan 1,3-β-glucosidase (EC 3.2.1.58); licheninase (EC 3.2.1.73); ABA-specific β-glucosidase (EC 3.2.1.175); β-1,3-glucanosyltransglycosylase (EC 2.4.1.-)	β-D-Glcp	Exo/**Endo**
**GH26**	**β-mannanase** (EC 3.2.1.78); exo-β-1,4-mannobiohydrolase (EC 3.2.1.100); β-1,3-xylanase (EC 3.2.1.32); **lichenase / endo-β-1,3-1,4-glucanase** (EC 3.2.1.73); mannobiose-producing exo-β-mannanase (EC 3.2.1.-)	β-D-Glcp; β-D-Manp; β-D-Xylp	Exo/**Endo**
**GH30**	**endo-β-1,4-xylanase** (EC 3.2.1.8); β-glucosidase (3.2.1.21); β-glucuronidase (EC 3.2.1.31); β-xylosidase (EC 3.2.1.37); β-fucosidase (EC 3.2.1.38); glucosylceramidase (EC 3.2.1.45); β-1,6-glucanase (EC 3.2.1.75); glucuronoarabinoxylan endo-β-1,4-xylanase (EC 3.2.1.136); endo-β-1,6-galactanase (EC:3.2.1.164); [reducing end] β-xylosidase (EC 3.2.1.-)	β-D-Glcp; β-D-Galp; β-D-Xylp; β-D-Fucp	Exo/**Endo**
**GH35**	**<u>β-galactosidase</u>** (EC 3.2.1.23); exo-β-glucosaminidase (EC 3.2.1.165); exo-β-1,4-galactanase (EC 3.2.1.-); β-1,3-galactosidase (EC 3.2.1.-)	β-D-Glcp; β-D-GlcNp; β-D-Galp	**Exo**
**GH39**	α-L-iduronidase (EC 3.2.1.76); **β-xylosidase** (EC 3.2.1.37).	α-L-IduAp, β-D-Xylp	**Exo**
**GH42**	**<u>β-galactosidase</u>** (EC 3.2.1.23); α-L-arabinopyranosidase (EC 3.2.1.-)	β-D-Galp; α-L-Arap	**Exo**
**GH50**	**β-agarase** (EC 3.2.1.81).	β-D-Galp	**Endo**
**GH51**	endoglucanase (EC 3.2.1.4); endo-β-1,4-xylanase (EC 3.2.1.8); **β-xylosidase** (EC 3.2.1.37); **α-L-arabinofuranosidase** (EC 3.2.1.55)	β-D-Glcp; β-D-Xylp; α-L-Araf	**Exo/Endo**
**GH53**	**endo-β-1,4-galactanase** (EC 3.2.1.89).	β-D-Galp	**Endo**
**GH59**	β-galactosidase (EC 3.2.1.23); **<u>β-galactocerebrosidase</u>** (EC 3.2.1.46)	β-D-Galp	**Exo**
**GH72**	**β-1,3-glucanosyltransglycosylase** (EC 2.4.1.-)	β-D-Glcp	**Endo**
**GH79**	β-glucuronidase (EC 3.2.1.31); **hyaluronoglucuronidase** (EC 3.2.1.36); **heparanase** (EC 3.2.1.166); baicalin β-glucuronidase (EC 3.2.1.167); β-4-O-methyl-glucuronidase (EC 3.2.1.-)	β-D-GlcAp	**Exo**/Endo
**GH86**	**β-agarase** (EC 3.2.1.81); β-porphyranase (EC 3.2.1.178)	β-D-Galp	**Endo**
**GH113**	**β-mannanase** (EC 3.2.1.78)	β-D-Manp	**Endo**
**GH128**	**β-1,3-glucanase** (EC 3.2.1.39)	β-D-Glcp	**Endo**

SI. 2. Puckering distributions of the residue at subsite -1 of β-galactosidases in clan GH-A during MD simulations of 100 ns (indicated) or 20 ns (by default). Mercator projections of the CP spheres for each of the ligands in this study are shown for each studied GH family and selected PDB and substrate pairs.

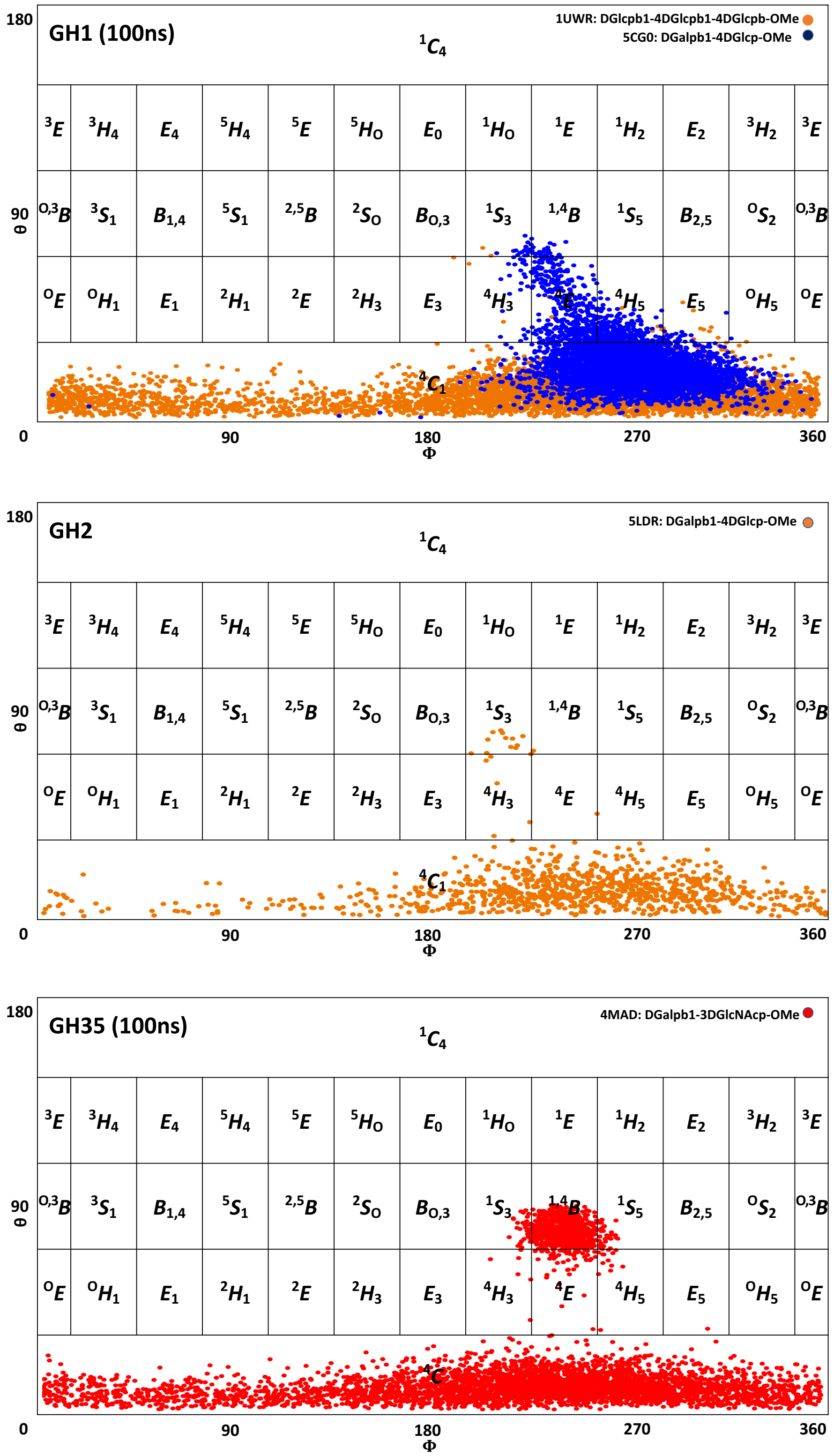

SI. 2 (cont.)

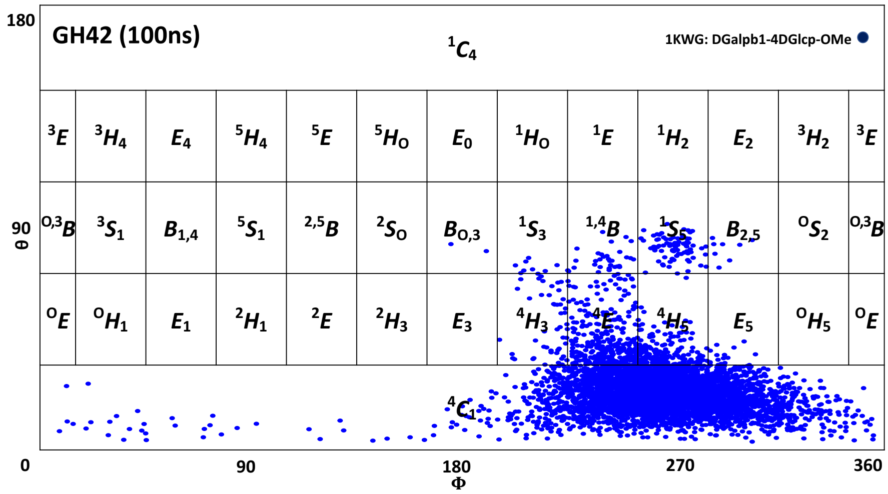
GH42 (100ns)
1KWG: DGalpb1-4DGlcp-OMe
$^1C_4$
^3E $^3H_4$ E_4 $^5H_4$ ^5E ^5H_O E_O ^1H_O ^1E $^1H_2$ E_2 $^3H_2$ ^3E
$^{O,3}B$ $^3S_1$ $B_{1,4}$ $^5S_1$ $^{2,5}B$ ^2S_O $B_{O,3}$ $^1S_3$ $^{1,4}B$ $^1S_5$ $B_{2,5}$ $^OS_2$ $^{O,3}B$
^OE $^OH_1$ E_1 $^2H_1$ ^2E $^2H_3$ E_3 $^4H_3$ ^4E $^4H_5$ E_5 $^OH_5$ ^OE
$^4C_1$
180
90
θ
0
90
180
270
360
Φ

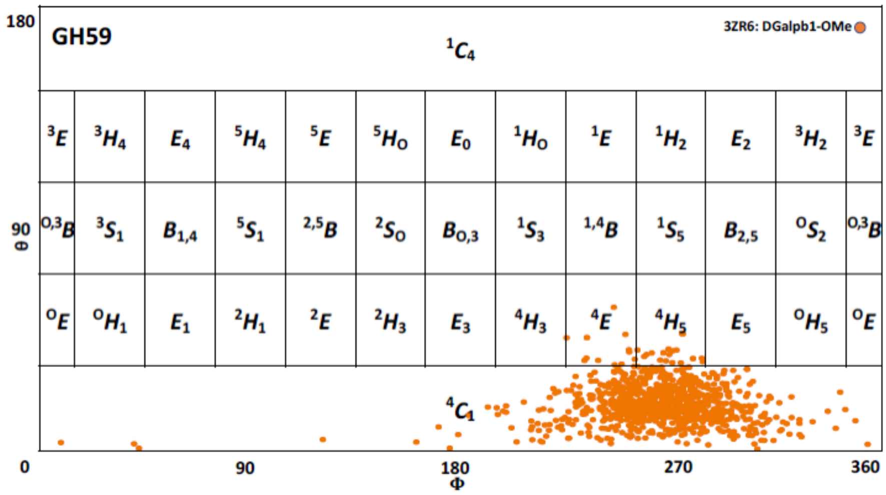
GH59
3ZR6: DGalpb1-OMe
$^1C_4$
^3E $^3H_4$ E_4 $^5H_4$ ^5E ^5H_O E_O ^1H_O ^1E $^1H_2$ E_2 $^3H_2$ ^3E
$^{O,3}B$ $^3S_1$ $B_{1,4}$ $^5S_1$ $^{2,5}B$ ^2S_O $B_{O,3}$ $^1S_3$ $^{1,4}B$ $^1S_5$ $B_{2,5}$ $^OS_2$ $^{O,3}B$
^OE $^OH_1$ E_1 $^2H_1$ ^2E $^2H_3$ E_3 $^4H_3$ ^4E $^4H_5$ E_5 $^OH_5$ ^OE
$^4C_1$
180
90
θ
0
90
180
270
360
Φ

SI. 3. Puckering variation with time observed at subsite -1 during MD simulations in families GH1, GH2, GH35 and GH42. Full 100 or 20 ns time segments that display conformational itineraries going from basal $^{4}C_{1}$ up to equatorial boat (*B*) and skew-boat (*S*) states are observed: (a) 1-methyl-β-lactoside = Lac-OMe (GH1, PDB ID: 5CGO); (b) 1-methyl-β-lactoside = Lac-OMe (GH2, PDB ID: 5LDR); (c) β-1,3-LacNAc-OMe (GH35, 4MAD); (d) 1-methyl-β-lactoside = Lac-OMe (GH42, PDB ID: 1KWG).

(a) GH1 – 5CGO:Lac-OMe

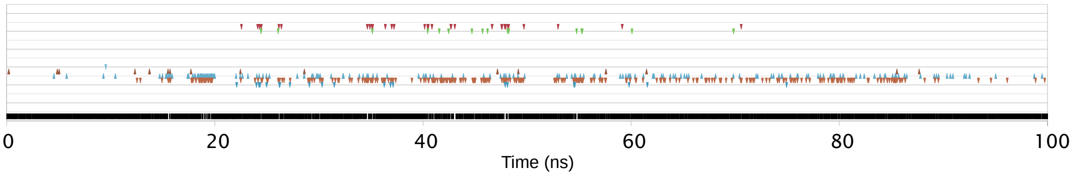

(b) GH2 – 5LDR:Lac-OMe

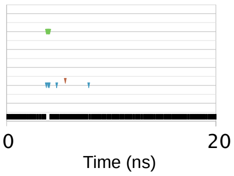

(c) GH35 – 4MAD:β-1,3-LacNAc-OMe

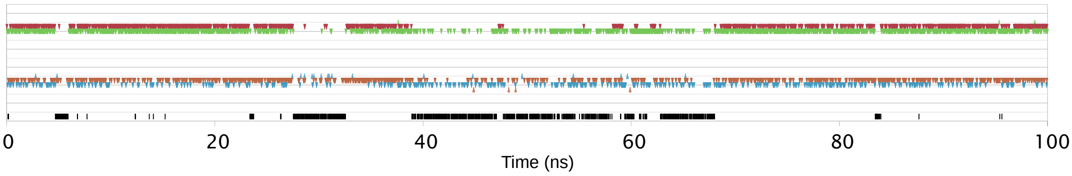

(d) GH42 – 1KWG:Lac-OMe

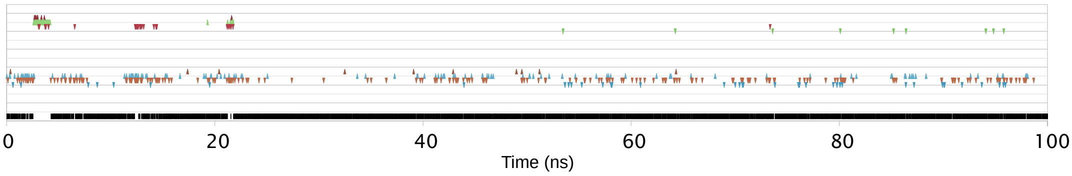

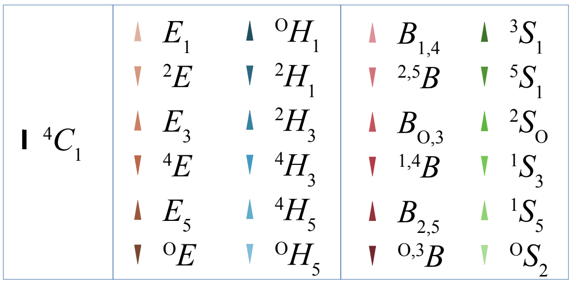

Supplement: Supplementary file 1 — Additional information [file 41598_2019_46589_MOESM1_ESM.pdf]
